# Dissecting the Non-Immune Tumor Microenvironment in Triple-Negative Breast Cancer: Molecular Subtype-Specific Patterns and Prognostic Implications

**DOI:** 10.3390/ijms262211211

**Published:** 2025-11-20

**Authors:** Antonia Syrnioti, Eleni Timotheadou, Vasileios Papadopoulos, Georgia Syrnioti, Triantafyllia Koletsa

**Affiliations:** 1 Department of Pathology, School of Medicine, Aristotle University of Thessaloniki, 54124 Thessaloniki, Greece; tonia.syrt@gmail.com; 2 Department of Medical Oncology, Papageorgiou University General Hospital, School of Medicine, Aristotle University of Thessaloniki, 56403 Thessaloniki, Greece; timotheadou@auth.gr; 3 First Department of Surgery, Papageorgiou University General Hospital, School of Medicine, Aristotle University of Thessaloniki, 56403 Thessaloniki, Greece; papadvas@auth.gr; 4 One Brooklyn Health, Brookdale University Medical Center, Brooklyn, NY 11212, USA; gsyrniot@bhmcny.org

**Keywords:** histology, immunohistochemistry, gene expression, progression, cancer-associated fibroblasts, extracellular matrix, angiogenesis, epithelial–mesenchymal transition, metabolic reprogramming, hypoxia

## Abstract

Triple-Negative Breast Cancer (TNBC) encompasses a biologically heterogeneous group of tumors, which can be classified into distinct molecular subtypes, namely basal-like 1 (BL1), basal-like 2 (BL2), immunomodulatory (IM), mesenchymal (M), mesenchymal stem-like (MSL), and luminal androgen receptor (LAR), with unique clinical and pathological characteristics. While immune features of these subtypes have been extensively characterized, the integration of non-immune stromal and structural components into our understanding of TNBC biology is only now being fully recognized. This narrative review synthesizes current evidence regarding differences in the non-immune microenvironment across TNBC molecular subtypes, with a focus on cancer-associated fibroblasts (CAFs), vascular features, extracellular matrix (ECM) dynamics, and epithelial–mesenchymal transition (EMT), along with metabolic–hypoxic reprogramming. Data from several studies are integrated to highlight subtype-specific signatures. Differences in stromal architecture and metabolic adaptations, potentially reflecting the underlying molecular heterogeneity, may hold prognostic or predictive significance and could inform personalized therapeutic strategies targeting the tumor–stroma interface.

## 1. Introduction

Triple-negative breast cancer (TNBC) represents approximately 15–20% of all breast carcinomas and is defined by the absence of immunohistochemical expression of estrogen receptor (ER) and progesterone receptor (PR), as well as the lack of HER2 protein overexpression or *HER2* gene amplification [1]. Clinically, TNBC is associated with early recurrence and metastatic spread, and limited targeted treatment options, leading to a generally poor prognosis [1]. Histologically, most TNBCs are high-grade invasive breast carcinomas of no special type (IBC, NST), whereas special histologic subtypes such as metaplastic, salivary gland-like, or apocrine carcinomas are less common [2]. TNBC may occur sporadically or in the context of hereditary cancer predisposition, most commonly involving germline *BRCA1/2* mutations [3]. Despite being grouped under a single label, TNBC seems to be a biologically and molecularly heterogeneous disease, arising from genomic instability, frequent *TP53* mutations, and defects in DNA repair pathways [4,5]. Multiple transcriptomic classifications have revealed distinct molecular subtypes. The seminal classification by Lehmann et al. identified six TNBC subtypes: basal-like 1 (BL1), basal-like 2 (BL2), immunomodulatory (IM), mesenchymal (M), mesenchymal stem-like (MSL), and luminal androgen receptor (LAR) [6]. Alternative systems, such as those proposed by Burstein et al. and Liu et al., converged on similar subtype groupings, further validating the biological diversity within TNBC [7,8].

Each molecular subtype exhibits unique gene expression patterns and biological traits, ranging from heightened proliferative activity in BL1, metabolic and growth factor signaling in BL2, and immune activation in IM to epithelial–mesenchymal transition (EMT) and stemness features in M and MSL subtypes. LAR tumors, on the other hand, are enriched for hormonally regulated and metabolically active gene programs, including androgen receptor (AR) signaling and lipid metabolism [9,10]. These molecular subtypes also tend to display characteristic histological patterns; for example, basal-like tumors frequently display high histologic grade and tumor necrosis [11]. IM tumors are often associated with prominent lymphocytic infiltration and myeloid-like morphology, M and MSL subtypes may show metaplastic features, while LAR tumors frequently exhibit apocrine morphology [11,12]. These intrinsic differences not only shape therapeutic responsiveness, such as improved chemosensitivity in BL1 and possible benefit from AR inhibition in LAR, but also suggest that the tumor microenvironment (TME) may vary substantially across subtypes [13,14,15]. While the immune composition of the TNBC TME has garnered increasing attention due to its prognostic and therapeutic relevance, especially in the context of immunotherapy [16], the non-immune components of the microenvironment remain comparatively underexplored. Non-immune elements such as cancer-associated fibroblasts (CAFs), vascular structures, adipose tissue, extracellular matrix (ECM) components, and metabolic gradients play critical roles in creating a complex and dynamic ecosystem, with a direct impact on tumor growth, invasion, therapeutic resistance, and metastatic potential [17,18,19,20]. These stromal and structural features are not passive bystanders but active modulators of tumor biology and may differ meaningfully between TNBC molecular subtypes.

This narrative review aims to synthesize available evidence on the heterogeneity of the non-immune TME across TNBC subtypes. We explore subtype-specific patterns in fibroblastic stroma, vascular density, ECM composition and stiffness, epithelial–mesenchymal transition (EMT), and metabolic or hypoxic adaptations. We also discuss their potential prognostic or predictive value and highlight how this evolving understanding could inform personalized treatment strategies in TNBC.

## 2. Results

Overall, published data on the non-immune components of the TME in TNBC remain relatively scarce, with available studies often limited in number and scope. Nevertheless, the analysis of non-immune components of the TME in TNBC reveals distinct patterns across molecular subtypes, encompassing CAFs, vascular structures, ECM remodeling, and EMT, as well as metabolic reprogramming. These features correlate with biological behavior and prognosis, offering potential therapeutic implications.

### 2.1. Cancer-Associated Fibroblasts (CAFs)

CAFs show pronounced variability among TNBC subtypes. M and LAR tumors have been reported to exhibit a high abundance of CD10^+^/GPR77^+^ CAF, a functionally distinct subset that has been linked to therapy resistance and unfavorable clinical outcomes [21,22]. LAR tumors, particularly, have also been reported to exhibit elevated levels of both myofibroblast-like (myCAFs) and inflammatory CAFs (iCAFs) [23]. Notably, among patients with this subtype, those achieving pathological complete response (pCR) after neoadjuvant chemotherapy (NAC) demonstrate a dynamic shift in CAF composition, characterized by a decrease in myCAFs and an increase in iCAFs, possibly reflecting therapy-related stromal remodeling [23]. A single-cell transcriptomic analysis further indicated that M and BL2-like tumors contain higher overall CAF abundance than LAR-like tumors [24]. By contrast, larger bulk transcriptomic analyses generally report BL tumors as comparatively stroma-low, with lower CD10^+^/GPR77^+^ CAF abundance, along with lower overall stromal signatures than M or LAR subtypes [21], highlighting potential subtype- and method-related discrepancies. Finally, the IM subtype consistently shows minimal CAF enrichment, aligning with its overall low stromal content [21].

### 2.2. ECM Remodeling and EMT Pathway

The ECM remodeling and EMT pathways further distinguish TNBC molecular subtypes in terms of invasiveness and stromal interaction. Specifically, consistent with earlier transcriptomic analyses by Lehmann et al., both the M and MSL subtypes are characterized by coordinated upregulation of motility, ECM organization, and EMT genes, features that collectively support a more invasive, stromally active phenotype [9,24,25,26]. In MSL tumors, this is also reflected in the upregulation of TGF-β signaling, as well as enhanced matrix metalloproteinase (MMP) expression [25,27]. Although such features have not been explicitly reported in the IM subtype, the β-catenin signaling pathway, which is known to influence not only immune responses but also structural processes like fibroblast activation and ECM deposition in other contexts, has been shown to be enriched in this subtype [26,28]. Additionally, the lncRNA LINC00312, which is downregulated in IM tumors, has been linked to β-catenin and PI3K/AKT pathways, both of which may intersect with EMT and ECM-related programs; though its direct role in ECM remodeling remains undefined [26,29,30]. Consistent enrichment of ECM or EMT signatures has generally not been described in BL and LAR tumors. In LAR, however, stromal signatures are elevated, likely reflecting CAF-driven remodeling [21,23,25].

### 2.3. Vascular Remodeling

Vascular components also differ markedly across TNBC subtypes. Mesenchymal and BL2 tumors show increased endothelial cell content, reflecting more developed vascular networks that likely support their proliferative and invasive behavior [24]. In the LAR subtype, endothelial cell abundance is generally lower [24]; however, one study using transcriptomic deconvolution reported that patients achieving pCR showed increased endothelial cell content, suggesting a possible link between vascular remodeling and therapeutic efficacy [23]. Concurrently, lymphangiogenesis—a separate but related axis of vascular remodeling—is elevated in both MSL and IM tumors, with the strongest enrichment observed in MSL [21]. In contrast, BL and M tumors exhibit low levels of lymphangiogenesis, further reinforcing the notion that vascular and lymphatic networks are heterogeneously distributed and subtype-specific within TNBC [21]. Finally, the IM subtype demonstrates a negative association with angiogenesis-inducing signatures [21]. Importantly, no clear data regarding pericyte abundance or subtype-specific distribution were identified in the available literature, indicating this remains an unexplored aspect of vascular heterogeneity in TNBC.

### 2.4. Metabolic & Hypoxic Reprogramming

Metabolic reprogramming further differentiates the non-immune TME among TNBC subtypes. LAR, M, and MSL tumors have been reported to exhibit high metabolic activity, particularly in fatty acid metabolism and adipogenesis [10,21,25]. Notably, MSL tumors have shown aberrant expression of adipocyte-associated genes, whereas this has not been reported in LAR tumors [10,25]. Interestingly, one study found that LAR tumors are enriched in glycolysis, while another observed that LAR tumors achieving pCR following neoadjuvant chemotherapy show reduced expression of glycolysis-related genes, suggesting a therapy-induced metabolic shift [23,25]. BL tumors are also enriched in metabolic processes, indicating a metabolically active phenotype [21]. IM tumors exhibit the lowest metabolic activity among TNBC subtypes, showing a negative association with glycolysis, lipid metabolism, and the pentose phosphate pathway [21]. Regarding hypoxia, LAR and M tumors have shown significant enrichment in hypoxia-related gene expression, while BL and IM tumors exhibit low hypoxia signatures, suggesting subtype-specific adaptation to the TME [21].

### 2.5. Cancer-Associated Adipocytes

Beyond general metabolic rewiring, the presence of cancer-associated adipocytes also appears to vary between TNBC subtypes. Specifically, MSL tumors uniquely demonstrate enrichment in adipocytes [25]. In contrast, LAR and M tumors exhibit strong lipid metabolic signatures in the absence of adipocyte enrichment, while BL and IM tumors show minimal or no evidence of adipose involvement within the TME [21,25].

**Figure 1:**
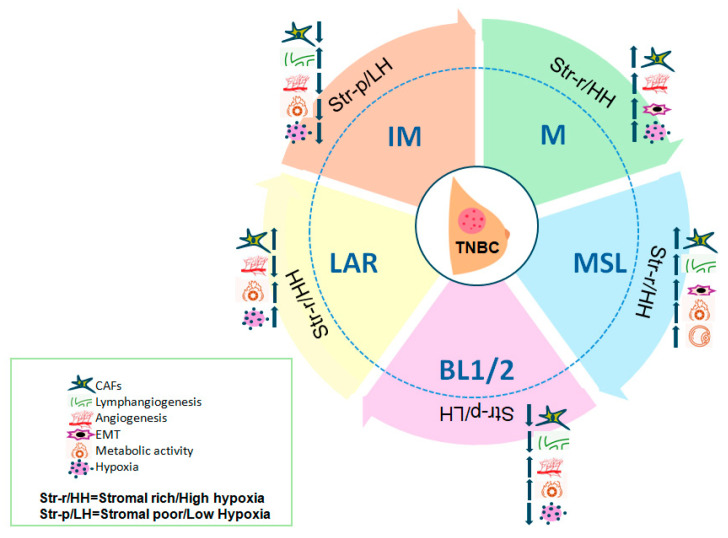
Molecular subtypes of triple-negative breast cancer (TNBC) and schematic overview of non-immune tumor microenvironment (TME) components.

A summary of the main non-immune tumor microenvironment characteristics across TNBC molecular subtypes is presented in Figure 1 and Table 1.

## 3. Discussion

Our analysis demonstrates that stromal, vascular, and metabolic elements of the TME display molecular subtype-specific patterns that may shape tumor behavior and therapeutic vulnerability. Specifically, IM tumors, with low CAFs [21], limited angiogenesis [21], and low metabolic activity [21], present a less stromally restrictive environment. Reduced CAF density has been associated with improved immune cell infiltration and potentially better drug penetration [31,32,33,34]. At the same time, minimal yet potentially functional angiogenesis may prevent the development of structurally aberrant vasculature, thereby limiting hypoxia and facilitating T-cell trafficking and function [35], while low glycolytic and lipid metabolic activity might reduce competition with infiltrating immune cells and decrease immunosuppressive lactate accumulation [36]. Beyond angiogenesis, lymphangiogenesis may also play a role in lymphocyte-rich tumors. Specifically, newly formed lymphatic vessels have been shown to promote antigen trafficking and T cell migration [37], which could potentially reinforce the immune-active phenotype of IM tumors. However, lymphatic endothelial cells may also exert immunosuppressive effects via various mechanisms such as MHC Class II antigen presentation to Tregs, high PD-L1 expression, or secretion of tolerogenic mediators [38,39]. Histologically, IM tumors often show marked lymphocytic infiltration within the stroma and are frequently accompanied by medullary features, aligning with their “immune-hot” phenotype [6]. These combined traits contribute to its enhanced sensitivity to immunotherapy and generally more favorable prognosis [13,21].

BL tumors are metabolically active [21], and show enrichment in endothelial cells [24]. Evidence regarding their stromal content is mixed: while single-cell analyses suggest that BL2-like tumors may harbor relatively high CAF abundance [24], larger bulk transcriptomic studies generally classify BL tumors as stroma-low, with reduced CD10^+^/GPR77^+^ CAF abundance and overall stromal signatures compared to M or LAR [21]. Importantly, BL tumors also exhibit low hypoxia [21], and lack consistent ECM/EMT activation. Although endothelial cell enrichment could promote tumor growth, the absence of a dense, fibrotic stroma and the low hypoxia may contribute to the generally favorable chemosensitivity observed in BL tumors [13].

By contrast, M and MSL tumors exhibit a highly stromal and angiogenic microenvironment, with abundant CAFs and endothelial cell enrichment [21,24]. ECM remodeling and EMT pathways are strongly activated, particularly in MSL tumors, with upregulation of TGF-β signaling and MMP expression [24,25]. These features have been repeatedly associated with limited drug penetration, chemoresistance, and enhanced invasive potential in TNBC [40]. Both subtypes also exhibit increased fatty acid metabolism and adipogenesis [21,25], which may also play a role in disease progression and even therapeutic resistance [41,42]. Notably, MSL tumors are further distinguished by the enrichment in cancer-associated adipocytes, which have been shown to induce EMT in breast cancer cells [25,43]. M tumors additionally demonstrate significant hypoxia-related gene expression [21], a feature known to drive EMT, stimulate angiogenesis, and foster an immunosuppressive TME [44,45,46]. Concurrently, MSL tumors show increased lymphangiogenesis [21], which may facilitate nodal and distal metastatic spread. Collectively, this fibrotic, angiogenic, and metabolically adaptive TME underlies the aggressive clinical course of these subtypes [13] and may support the rationale for therapies targeting the tumor–stroma interface, including TGF-β or fibroblast activation protein-α (FAP) inhibition, ECM modulation, and anti-angiogenic strategies—potentially in combination with chemotherapy or immunotherapy [47,48,49]. Beyond abundance, CAF functional states influence the microenvironment via cytokine secretion (notably IL-6 and CXCL12/SDF-1), which modulate immune cell recruitment [31,50]. These paracrine signals, acting alongside TGF-β, may induce LOX expression and fibronectin fibrillogenesis, increasing ECM crosslinking and stiffness and thereby reinforcing pro-tumorigenic EMT [31,50]. Such mechanisms may be particularly relevant to the fibrotic microenvironment observed in M/MSL and to the CAF-rich stroma of LAR tumors.

LAR tumors harbor CD10^+^/GPR77^+^ CAFs, which have been linked to poor overall survival [21] and dual enrichment in myCAFs and iCAFs [23]. Interestingly, in patients achieving pCR after NAC, LAR tumors exhibit a therapy-induced stromal shift, with decreased myCAFs and increased iCAFs, suggesting that this stroma is dynamically reprogrammable [23]. Metabolically, LAR tumors are characterized by elevated fatty acid metabolism and glycolytic activity [21,25], which may enable adaptation to nutrient stress and promote therapy resistance and metastatic potential [51,52]. Beyond metabolic reprogramming, recent mechanistic evidence indicates that co-targeting the AR and vitamin D receptor (VDR) pathways synergistically suppresses TNBC proliferation and invasion, suggesting a combined AR/VDR therapeutic vulnerability that may be particularly relevant for this subtype [53]. pCR cases have been further noted to exhibit reduced glycolysis, indicating that metabolic reprogramming accompanies effective therapy [16]. Endothelial cell content increases in responders [23], while a significant enrichment in hypoxia-related gene expression has also been reported [21]. Overall, this CAF--rich but therapeutically reprogrammable stroma, combined with hypoxia--driven metabolic flexibility, highlights the potential of targeting metabolic vulnerabilities, such as fatty acid oxidation or hypoxia-adaptive signaling [45,54], in combination with AR-directed or standard chemotherapeutic approaches.

In addition to stromal, vascular, and metabolic influences, hormonal and inflammatory signaling also contribute to shaping the TNBC microenvironment. The AR and estrogen receptor β (ERβ) pathways can modulate stromal remodeling and metabolic adaptation, while inflammatory mediators such as IL-6, TNF-α, and NF-κB promote fibroblast activation and extracellular matrix reorganization [55,56,57,58]. These intersecting signals may indirectly influence the subtype-specific TME patterns.

Despite emerging insights, significant gaps remain in our understanding of the non-immune TME across TNBC molecular subtypes. Although this review integrates multiple studies to delineate stromal–immune interactions across TNBC subtypes, heterogeneity among datasets and experimental approaches may limit the generalizability of some observations. For example, reported discrepancies in CAF abundance—higher in M and BL2-like tumors in single-cell datasets but lower in bulk transcriptomic analyses—likely reflect methodological differences such as sample size, tumor region sampling, and the averaging effects of transcriptomic deconvolution [21,24]. Single-cell studies offer superior cellular resolution but often analyze few cases and may overrepresent localized stromal niches, whereas bulk datasets provide statistical power at the expense of spatial context. Similar methodological biases may underlie inconsistencies in vascular or metabolic profiling across studies. Overall, transcriptomic approaches—whether bulk or single-cell— are seldom complemented by histologic or functional validation, leaving key stromal components insufficiently characterized.

To address these limitations, future analyses of patient-derived samples should incorporate integrated spatial-omics and single-cell approaches coupled with histologic validation. Particular attention should be given to pericyte coverage and heterogeneity using established markers, such as PDGFRβ, desmin, NG2, and αSMA, to determine subtype-specific patterns [59]. Likewise, other underexplored aspects, such as ECM mechanotransduction, nerve–tumor interactions or the spatial heterogeneity of stromal elements between tumor core and invasive front, warrant systematic investigation to capture the full complexity of the TNBC microenvironment [60,61]. Recent spatial transcriptomic studies have revealed distinct perivascular niches and mechanosensitive ECM signatures influencing therapy response in breast and other solid tumors, underscoring the potential of such high-resolution mapping to advance understanding of TNBC stromal biology [62,63]. Future work incorporating spatial multi-omics and longitudinal sampling before and after therapy will be crucial to validate dynamic changes within the tumor microenvironment and to identify clinically actionable stromal biomarkers. Overall, addressing these limitations through integrated spatial, single-cell, and functional validation approaches will be essential to translate current molecular insights into clinically applicable biomarkers and therapeutic targets.

## 4. Materials and Methods

This narrative review synthesizes published evidence from 2011, when TNBC molecular subtyping was originally introduced [6], to 2025, focusing on studies that examined the non-immune tumor microenvironment in TNBC across molecular subtypes. Relevant literature was identified using PubMed, Scopus, and Web of Science biomedical databases, guided by keywords including “TNBC,” “molecular subtypes,” “tumor microenvironment,” “cancer-associated fibroblasts,” “vascular remodeling,” “microvascular density”, “angiogenesis”, “lymphangiogenesis” “pericytes”, “extracellular matrix,”, “epithelial–mesenchymal transition”, “hypoxia,” “metabolism”, and cancer-associated adipose cells. The selection of these keywords was guided by widely accepted hallmarks of the tumor microenvironment [64], ensuring coverage of structural, stromal, metabolic, and vascular processes central to TNBC progression. Studies were prioritized if they provided histologic, immunohistochemical, or transcriptomic insights into key structural components of the TME.

## 5. Conclusions

In conclusion, the non-immune TME of TNBC is highly heterogeneous and molecular subtype-specific, encompassing distinct stromal, vascular, and metabolic ecosystems that interact with tumor biology and influence clinical outcomes. Incorporating these features into clinical and translational research could refine prognostication and pave the way for stroma-targeted, metabolism-informed, and combination therapeutic strategies in TNBC. Ultimately, integrating spatial and functional characterization of these components across subtypes will be essential to translate current insights into personalized treatment approaches and improved patient outcomes.

## Figures and Tables

**Table 1:** Summary of Non-Immune TME Features Across TNBC Molecular Subtypes.

Subtype	CAFs	Vascular Features	ECM/EMT Features	Metabolic Profile	Hypoxia
BL1/2	Stroma-low in bulk studies, though single-cell analyses suggest higher CAF abundance in BL2-like tumors	Increased endothelial cell content; Low levels of lymphangiogenesis	No consistent ECM/EMT enrichment	Metabolically active	Low hypoxia signatures
IM	Minimal CAF enrichment	Elevated lymphangiogenesis; Negative association with angiogenesis	No consistent ECM/EMT enrichment	Lowest metabolic activity; negative association with glycolysis, lipid metabolism, and PPP	Low hypoxia signatures
M	High CAF abundance	Increased endothelial cell content; Low levels of lymphangiogenesis	Pronounced ECM and EMT activation	Not directly reported	Enrichment in hypoxia-related gene expression
MSL	High CAF abundance	Elevated lymphangiogenesis	Pronounced ECM and EMT activation	High metabolic activity (esp. fatty acid metabolism and adipogenesis)	Not directly reported
LAR	High myCAFs and iCAFs (but lower than M or BL2); dynamic remodeling post-therapy	Low baseline endothelial cell content; increase with pCR	No consistent ECM/EMT enrichment, but CAF-driven remodeling possible	High metabolic activity (esp. fatty acid metabolism and adipogenesis); glycolysis decreases post-pCR	Enrichment in hypoxia-related gene expression

Abbreviations: TNBC, triple-negative breast cancer; TME, tumor microenvironment; CAFs, cancer-associated fibroblasts; myCAFs, myofibroblast-like cancer-associated fibroblasts; iCAFs, inflammatory cancer-associated fibroblasts; ECM, extracellular matrix; EMT, epithelial–mesenchymal transition; BL1/2, basal-like 1/2; IM, immunomodulatory; M, mesenchymal; MSL, mesenchymal stem-like; LAR, luminal androgen receptor; pCR, pathological complete response; PPP, pentose phosphate pathway.

## Data Availability

No new data were created or analyzed in this review. Data sharing is not applicable to this article.

## Abbreviations

The following abbreviations are used in this manuscript:

AR	Androgen receptor
CAF	Cancer-associated fibroblast
ECM	Extracellular matrix
EMT	Epithelial–mesenchymal transition
ER	Estrogen receptor
HIF	Hypoxia-inducible factor
IBC, NST	Invasive breast carcinoma of no special type
iCAF	Inflammatory cancer-associated fibroblast
IM	Immunomodulatory subtype
LAR	Luminal androgen receptor subtype
M	Mesenchymal subtype
MMP	Matrix metalloproteinase
MSL	Mesenchymal stem-like subtype
myCAF	Myofibroblast-like cancer-associated fibroblast
NAC	Neoadjuvant chemotherapy
pCR	Pathological complete response
PR	Progesterone receptor
TGF-β	Transforming growth factor beta
TME	Tumor microenvironment
TNBC	Triple-negative breast cancer
VDR	Vitamin D receptor
